# Medical Opinion and Movement

**Published:** 1909-12-18

**Authors:** 


### Medical Opinion and Movement.

EXCISION of the Scapula is an operation that few medical men are ever called upon to undertake, and for that reason any case which has been carefully studied is worthy of publication. In the *Australasian Medical Gazette*, Drs. Marten and Gilbert describe very fully their procedure when confronted by a sarcoma of the infra-spinatus muscle invading the bone. They adopted the principles laid down in Cheyne and Burghard's Surgery, and practised them first on the cadaver. They began by opening the axilla and ligaturing the sub-scapular vessels: this step they approve very strongly. Then, following Cheyne and Burghard, they passed a finger up the axilla to the outer side of the main vessels, and endeavoured with scissors to free the coracoid process from its attachments. In this, as it turned out, they failed, and at a later stage of the operation they were obliged to cut through the base of the process with bone forceps, and leave it *in situ*. They hold that, unless the growth is invading the axilla or the coracoid process, it is a mistake to remove this part of the scapula; and accordingly they would modify the textbooks on this point. The operation was concluded on classic lines, and their only other criticism is that the period of drainage usually advised is too short, owing to the free discharge of serum from the enormous area of the wound.

A PLEA for the recognition in textbooks of surgery, or at least in those of tropical diseases, of the characters of Traumatic Lesions due to Wild Beasts, is advanced in the *Journal of Tropical Medicine and Hygiene*, by Dr. R. Howard, who has had extensive experience of such injuries in Nyasaland. While the author's remarks on the characteristics which distinguish the bites or scratches of one animal from those of another are interesting, it does not appear that there is any special departure from the established principles of antiseptic surgery which he would recommend; and therefore the need for a special literature is not very obvious. Leopards he finds one of the most dangerous of these wild animals, an experience which is corroborated by that of most others who have dwelt in savage Africa. This animal uses teeth and claws simultaneously, and as a rule devotes most attention to the neck: consequently injuries to the great nerves and vessels are common, and Dr. Howard estimates the primary mortality at about fifty per cent. Patients have, as a rule, not less than ten wounds, and often more: the claw marks are most deceptive, a narrow slit often covering a long curving track through which virulent organisms have been transferred deeply into the cellular planes of the neck. Free and careful syringing with antiseptic lotions, and efficient drainage with tubes, are essential. Lion wounds are much larger, and consequently drain with less difficulty: compound fractures are common. Hippopotami have been known to bite limbs clean off (so have lions, though Dr. Howard does not seem to know it), and crocodiles also inflict most extensive injuries. In all these cases the free use of strong antiseptics will save many lives: though it is not necessary to follow the example of one titled sportsman, who claims to have saved the life of a man after the scalp had been torn off by a leopard, by packing the whole wound with crystals of carbolic acid.

THE same author pleads for the use of pure quinine, the alkaloid, in the treatment of malaria. He was induced to try this because he found that the black babies who are brought for treatment to his clinic always spit out the quinine salts as soon as put into the mouth. His method is to use the alkaloid as a dry powder, not a solution: this he plants as far back on the tongue as possible, and the mother at once puts the child to the breast. The powder is non-crystalline, practically tasteless, is easy to dispense, and contains 95 per cent. of anhydrous quinine, a much larger proportion than that contained in any other quinine preparation. Though slightly more expensive than the usual salts, it is far less costly than euquinine; and it seems, according to the author's experience, to be highly effective. He has also tried it for adults, and there seems no reason why it should not receive, as he recommends, a more extended trial for those patients who object strongly to the taste of the ordinary fluid forms of quinine salts. Tannate of quinine has been tried, but abandoned on account of its low percentage of alkaloid and its uncertain composition.

A STATISTICAL Analysis of Sixty-eight Thousand Cases of Typhoid Fever is the gigantic task to which Dr. Sallom, of Philadelphia, is applying himself, being the number of cases that have occurred in Philadelphia during a period of eleven and a half years. A preliminary abstract is all that has been accomplished so far, though even then some interesting points have been brought to light. It is perhaps unlikely that a series such as this, gathered from the practice of a large number of physicians, will yield results as important as those obtained by Professor Osler and his assistants from the classical series they investigated at the Johns Hopkins Hospital; but there are some respects in which accuracy is not impaired, and in which the enormous number of cases excludes the chances of fallacy by the law of averages. Thus it seems clear that February, and not October or November, is the most typhoid-stricken month in Philadelphia; and this pre-eminence would, of course, be greater had it the usual number of days. January is the next most prolific month. The new and improved water supply of the city has but just been completed, and has not yet had time to affect the returns now analysed: but there seems reasons to believe that those quarters to which it was first extended showed a marked diminution in typhoid notifications at once. It is worthy of remark that a writer in the *Annals of Surgery*, in reporting a case of spontaneous rupture of the spleen during this disease, expresses his belief that it is much commoner than has been supposed, and that most of the cases go undiagnosed. It would be of interest to know whether Dr. Sallom can support this contention.

---

The Ætiology of Insular or Disseminated Sclerosis has long been as obscure as the symptoms of that protean disease are apt to be perplexing. A case which came to autopsy recently in Paris is the subject of a careful report by Guiéva-Rajes, who is a worker under Raymond at the Charcot clinic of the Salpêtrière. The patient died during an acute exacerbation of his disease, and in his brain and spinal cord were found not only the usual sclerosed plaques from which the French name of the disease has been taken, but also reddish, softer, less sharply defined patches. These, on microscopic examination, were seen to be definitely inflammatory, a leucocytic exudate being a prominent feature. The glia and the myelin sheath of the nerve fibres were extensively broken down, but the axis cylinders were comparatively unaffected. The extent and distribution of these zones corresponded roughly with those of the typical older patches of sclerosis, and the inference is that the newer plaques were responsible for the exacerbation of symptoms which the patient had exhibited. It seems at any rate reasonable to assume this, and also to argue in favour of a microbic origin for the disease: it is clear that this conception, if it turns out to be based on a solid foundation, may open up possibilities for the future of much greater success in the treatment of disseminated sclerosis than has ever been attained in the past.

---

A novel suggestion for the Cure of Hernia in Children is put forward, after practical experience, by Mr. A. G. Faulds in the *Glasgow Medical Journal*. This is nothing less than subcutaneous suture of the inguinal canals without any skin incision, which allows of the immediate removal of the patient to his home after the operation. He advocates the method for those cases of inguinal hernia in childhood where there is no complication such as hydrocele, and where there is no difficulty in defining the pillars of the external ring. The patient is anæsthetised and the skin prepared for operation in the usual way. The surgeon then reduces the hernia, and introduces his left forefinger into the canal by invaginating the scrotum. He thus feels both pillars of the ring, and pierces the skin about the top of the opening with a strong curved needle armed with stout silk. As the needle is made to pierce the pillar of the ring, he is able to feel it with the forefinger, and to ensure that the gut remains reduced and out of harm's way. He then guides the needle to the opposite pillar, and, taking a good bite of it, brings the needle point out through the skin. Reintroducing it at the same place, he then directs the needle into the subcutaneous fat down the long axis of the canal, again pierces the pillar of that side in the reverse direction to the previous stitch, carries the needle across the ring once more and through the pillar first attacked, and then brings it out by travelling up the subcutaneous fat to the original point of entrance. A purse string suture is thus obtained, which must be pulled very tight and tied. The knot can easily be buried altogether, so that the suture is never removed. Adhesions form in the hernial sac, and nature completes the cure. The after-results are described as highly satisfactory.

---

At the end of a most instructive paper on Temperatures as Studied in Sanatoria, which appears in the *Magazine of the London School of Medicine for Women*, from the pen of Miss C. Colley, are some remarks on the subject of the normal premenstrual rise and an appeal for charts of healthy persons for the investigation of this phenomenon. When the temperature is taken with the exact care as to every detail of time, atmospheric environment, and relation to meals and exercise, as is done in sanatoria, it is found that in nearly every female patient there is a rise, beginning as a rule about fourteen days before the onset of menstruation. The rise is at first sudden, and then mounts by smaller increments until it may reach 99.6 to 100 in a patient whose temperature, as far as disease is concerned, is normal. After a few days at this maximum, it falls gradually until the day before menstruation, when it frequently falls suddenly to subnormal. Sometimes there are slight rises on the evenings of the second or third day of menstruation, but more commonly the normal is maintained until the next cycle begins. In cases with slight pyrexia the same curve can be demonstrated, superimposed on that due to the tubercle bacillus; but in more advanced cases the fluctuations are too great for the detection of this factor. Miss Colley appeals for help to decide whether this rise exists in healthy women, or whether it is a sanatorium phenomenon; and also whether it can be detected during pregnancy, after the menopause, and before puberty. She gives in detail the precautions essential to the determination of the body temperature with the accuracy necessary for this purpose.

---

A cutaneous Reaction for the Diagnosis of Typhoid Fever is described in the *University of Pennsylvania Medical Bulletin* by Dr. S. J. Dechan. The reaction can frequently be obtained several days before the Widal, and is complete in twenty-four hours without any ill-effects to the patient; it is not found in any other disease except typhoid. A virulent strain of typhoid bacilli is grown on slabs for twenty-four hours. The organisms are then washed free of the medium with saline solution, emulsified, and incubated four days at 37.5° C. They are then sterilised at 60° C., centrifugalised, and the clear supernatant liquid removed with a pipette. This fluid, which must be sterile, is placed while fairly fresh on the patient's skin, which is then gently scarified without drawing blood. A control abrasion is made in sterile saline solution at a distance of one inch. Three grades of positive reaction are described. One is a small papule in the centre of a hyperæmic zone two to four millimetres in diameter. The next is a collection of papules in a hyperæmic and œdematous area of four to eight millimetres diameter. The third is an œdematous urticarial wheal, from which serum may exude, in a hyperæmic zone of two to three centimetres in width.

---

In last week's issue an account was given in these columns of the Treatment of Acne by means of the method of electrical ionization. Another form of treatment for this affection, which appears to give satisfactory results, consists in the application of the hot-air douche. An account of the treatment is given in the *Journal de Médecine de Paris* by Dr. Raoult Deslongchamps. He applies the hot-air douche at 120° C. for a few minutes to the part affected, and in the case of the face, of course, he takes necessary precautions to protect the eyes, nose, and mouth. Two or three applications are generally sufficient to effect a cure. The author records a case of a young actress, aged 26, who had been much troubled with a severe form of acne over the face and front part of the neck. The condition had persisted for ten years in spite of every other kind of treatment. The author gave a course of radiotherapy for five weeks, with the result that the pustules dried up and desquamated; but as soon as they disappeared a fresh crop developed. The author then tried the hot-air douche, with the result that after two applications the pustules completely disappeared without any further recurrence, and the patient has remained quite cured for some time. The author ascribes the effect to the increased flow of blood to the part which the hot-air douche produces. He has also obtained satisfactory results in the treatment of chronic superficial ulcerating wounds, such as varicose ulcers, tuberculous ulcers, and the like.

---

Apropos of the communication of Metchnikoff on the Relation between the Bacillus Proteus and Infantile Diarrhœa, already reported in these columns, Dr. Vincent gave an account, at a meeting of the *Académie de Médecine*, of two cases of severe gastro-enteritis in adults after the ingestion of pork butcher's scraps. In both cases he found the bacillus proteus in abundance in the vomit and stools of the patients. He has also found the same microbe in the stools of patients suffering from indigestion after eating salad and *pâté de foie faisandé*. He considered that the symptoms were due to the multiplication of the microbe in the digestive tract rather than to intoxication by the toxins already in the food. Under normal conditions the bacillus proteus is rarely found in the human intestines. It is carried thither on the food by the hands, flies, the receptacles of the food, and with the dust. He thinks that certain conditions ascribed to a paratyphoid infection are really due to infection with the bacillus proteus. The author further reported a case of mixed infection by the bacillus proteus and bacillus typhosus, in which the symptoms were of a severe type. Experimentally the association of these two micro-organisms is very pathogenic for animals.

A good deal of discussion has been taking place at Vienna on the treatment of Goître, and especially of Basedow's disease, by radiotherapy. At a meeting of physicians and scientists, Dr. Von Eiselsberg expressed the opinion that the efficacy of the treatment is very problematic; on the other hand, it causes adhesions and increased vascularity of the gland and surrounding parts, making ultimate operation much more difficult and dangerous. At a meeting of the Medical Society of Vienna Dr. Holzknecht took up the cudgels against this view. He recorded nine cases of exophthalmic goître in which he had obtained most favourable results from the treatment, and in which, although certain morbid signs such as exophthalmia and tremors persisted, he considered a virtual cure was effected. Furthermore, he had received similar favourable reports from Carl Beck, of New York; Görl, of Nuremberg; and Krause, of Bonn. In reply, Dr. Von Eiselsberg gave an account of three cases submitted to radiotherapy and eventually operated upon, in which he had encountered the difficulties mentioned above. Of 795 operations for goître, of which he had record in his hospital, only two had presented similar adhesions, and in both these cases radiotherapy had been previously practised. Several other speakers testified to this tendency to the formation of adhesions as a result of radiotherapy, and adopted a sceptical attitude in regard to the efficacy of the treatment. Dr. Chvostek expressed the opinion that, at any rate, the $x$-rays gave no better result than ordinary medicinal treatment, and reported a case in which ordinary goître developed into Basedow's disease after the application of the $x$-rays.

---

At a meeting of the Société de Chirurgie Dr. Psaltoff, of Smyrna, gave an account of a remarkable case of intestinal accumulation of fruit-stones. The patient was a young girl, aged 12, who complained of abdominal pain recurring about every 10 days and lasting a few hours. In the course of a few months these pains became more constant and violent, and were accompanied by fever and vomiting. On palpation of the abdomen an elongated tumour was felt in the right iliac fossa, sensitive to pressure, of a somewhat soft consistency and not adherent to the surrounding parts. Operative interference was decided upon. On opening the abdomen the last part of the small intestines, to the extent of 20 centimetres, was found to be distended to three times its normal size, and the walls were very thick and inflamed. On palpating this part, foreign bodies could be distinctly felt slipping under the fingers and producing a sort of crepitation. The intestine was incised and a large number of fruit-stones of all kinds (cherry, prune, plum, grape) were evacuated, altogether 794 stones. The patient made a rapid and complete recovery. From information obtained it appeared that the stones had been swallowed four years ago. The first lot of stones had collected in a diverticulum at the ilio-cæcal valve, and in turn these had obstructed the passage of those swallowed subsequently. It is remarkable that there were never any signs of actual intestinal obstruction.

